# Supplementary material for: Resting-state alterations in behavioral variant frontotemporal dementia are related to the distribution of monoamine and GABA neurotransmitter systems
Source: eLife. 2024 Jan 15;13:e86085. doi: 10.7554/eLife.86085 (PMC10789488; doi:10.7554/eLife.86085)
Supplement: Supplementary file 1. [file elife-86085-supp1.docx]

# Supplementary File 1

| **supplementary file 1a** Distribution of number of subjects, age and sex across centers. | | | | | | |
| --- | --- | --- | --- | --- | --- | --- |
| **Center** | **N** | | **Age (Mean ± Std.)** | | **Sex (male/female)** | |
|  | **HC** | **bvFTD** | **HC** | **bvFTD** | **HC** | **bvFTD** |
| Bonn | --- | 6 | --- | 66.7 ± 7.74 | --- | 6/0 |
| Erlangen | 1 | 4 | 74.0 ± 0.00 | 60.5 ± 4.65 | 1/0 | 3/1 |
| Göttingen | 1 | 5 | 37.0 ± 0.00 | 66.8 ± 7.43 | 0/1 | 5/0 |
| Homburg | 6 | 5 | 56.0 ± 10.5 | 59.8 ± 12.6 | 4/2 | 4/1 |
| Leipzig | 4 | 13 | 61.5 ± 8.23 | 61.8 ± 12.6 | 2/2 | 8/5 |
| München (TU) | --- | 13 | --- | 60.3 ± 8.87 | --- | 8/5 |
| Rostock | 8 | 4 | 73.4 ± 5.66 | 59.5 ± 9.54 | 2/6 | 2/2 |
| Tübingen | --- | 2 | --- | 48.5 ± 7.78 | --- | 2/0 |
| Ulm | 2 | --- | 59.5 ± 2.12 | --- | 0/2 | --- |
| Bonn – University of Bonn, German Center for Neurodegenerative Diseases (DZNE), University Hospital Bonn  Erlangen – University Hospital Erlangen  Göttingen – Medical University Göttingen  Homburg – Saarland University Hospital  Leipzig – Max-Planck-Institute for Human Cognitive and Brain Sciences  TU München – Technical University of Munich  Rostock – University Hospital Rostock, German Center for Neurodegenerative Diseases (DZNE)  Tübingen – University Hospital Tübingen, Centre for Neurology, Hertie-Institute for Clinical Brain Research  Ulm – Ulm University | | | | | | |

| **supplementary file 1b** Detailed neurotransmitter map information incl. tracer, number of subjects, age, sex and study. | | | | | |
| --- | --- | --- | --- | --- | --- |
| **Neurotransmitter** | **Tracer** | **N** | **Age** | **Sex ratio**  **(% male subjects)** | **Study (doi)** |
| 5-HT1a | way100635 | 35 | 26.3 ± 5.20 | 51.00 % | 10.1016/j.neuroimage.2012.07.001 |
| 5-HT1b | p943 | 23 | 28.7 ± 7.00 | 65.00 % | 10.1016/j.neuroimage.2012.07.001 |
| 5-HT2a | altanserin | 19 | 28.2 ± 5.70 | 58.00 % | 10.1016/j.neuroimage.2012.07.001 |
| 5-HTT | dasb | 18 | 30.5 ± 9.50 | 67.00 % | 10.1016/j.neuroimage.2012.07.001 |
| D1 | sch23390 | 13 | 33.0 ± 13.0 | 46.00 % | 10.1007/s00259-017-3645-0 |
| D2 | flb457 | 55 | 32.5 ± 9.69 | 47.27 % | 10.1038/jcbfm.2014.237 |
| DAT | fpcit | 174 | 61.0 ± 11.0 | 62.64 % | 10.1038/s41598-018-22444-0 |
| FDOPA | fluorodopa | 12 | 55.1 ± 16.6 | 50.00 % | 10.33588/imagendiagnostica.901.2 |
| GABAa | flumazenil | 6 | 43.0 ± 4.00 | 100.00 % | 10.1038/s41598-018-22444-0  10.1038/jcbfm.2011.177 (original study) |
| MU | carfentanil | 204 | 32.3 ± 10.8 | 64.71 % | 10.1038/mp.2017.183 |
| NET | mrb | 10 | 33.3 ± 10.0 | 60.00 % | 10.1007/s00259-016-3590-3 |

| **supplementary file 1c** Contrast peak voxels (HC>bvFTD) incl. MNI coordinates, corresponding anatomical region, t-value and cluster size for fALFF, fALFF additionally corrected for total GMV, and GMV. | | | | | | |
| --- | --- | --- | --- | --- | --- | --- |
|  | **MNI coordinates at peak voxel (mm)** | | | **Corresponding**  **anatomical region** | **T**  **(peak-level)*** | **Cluster size (voxels)** |
|  | **x** | **y** | **z** |  |  |  |
| fALFF | -42 | 6 | 18 | Left inferior frontal gyrus | 6.76 | 6973 |
|  | 39 | -72 | 45 | Right inferior lateral parietal lobe | 5.64 | 927 |
|  | -39 | -27 | 60 | Left postcentral gyrus | 5.17 | 310 |
|  | -54 | -18 | -6 | Left superior temporal gyrus | 4.39 | 113 |
|  | -18 | 3 | 24 | Left caudate nucleus | 4.34 | 70 |
|  | 9 | 6 | 0 | Right lateral temporal ventricle | 4.91 | 45 |
| fALFF (corrected for GMV) | -42 | 6 | 18 | Left inferior frontal gyrus | 5.11 | 331 |
|  | 57 | 21 | 0 | Right inferior frontal angular gyrus | 4.89 | 202 |
|  | 0 | -33 | 30 | Left posterior cingulate gyrus | 5.23 | 170 |
| GMV | -45 | -24 | 12 | Left superior temporal gyrus | 7.52 | 20308 |
| * cluster-level threshold: p-uncorrected = .001 with permuted cluster threshold (fALFF: 42 voxels, fALFF corrected for GMV: 42 voxels, GMV: 38 voxels)  **fALFF – fractional amplitude of low frequency fluctuations, GMV – gray matter volume** | | | | | | |

| **supplementary file 1d** Fisher’s z transformed Spearman correlations of neurotransmitter maps with fALFF, fALFF additionally corrected for total GMV and gray matter volume. | | | | | |
| --- | --- | --- | --- | --- | --- |
| **Neurotransmitter** | | | **fALFF** | **fALFF**  **(corrected**  **for GMV)** | **GMV** |
| 5-HT1a | r (Spearman) | | -.0316 | -.0223 | -.0787 |
|  | p | | .5637 | .6819 | .1484 |
| 5-HT1b | r (Spearman) | | -.2152* | -.1521* | -.1143 |
|  | p | | <.0001 | .0044 | .0597 |
| 5-HT2a | r (Spearman) | | -.1653* | -.1087 | -.0771 |
|  | p | | .0014 | .0378 | .2366 |
| 5-HTT | r (Spearman) | | -.0219 | -.0603 | -.0525 |
|  | p | | .7452 | .3612 | .4543 |
| D1 | r (Spearman) | | -.0613 | -.0656 | -.0660 |
|  | p | | .1603 | .1387 | .2924 |
| D2 | r (Spearman) | | -.0140 | -.0289 | -.0640 |
|  | p | | .7956 | .5852 | .3465 |
| DAT | r (Spearman) | | .0004 | -.0492 | -.0171 |
|  | p | | .9962 | .5012 | .7960 |
| FDOPA | r (Spearman) | | -.0225 | -.0558 | -.0144 |
|  | p | | .7389 | .4043 | .8311 |
| GABAa | r (Spearman) | | -.1256* | -.0783 | -.0589 |
|  | p | | .0149 | .1328 | .3928 |
| MU | r (Spearman) | | -.0676 | -.0664 | -.1104 |
|  | p | | .2807 | .2936 | .1879 |
| NET | r (Spearman) | | -.1353* | -.1192 | -.0116 |
|  | p | | .0157 | .0432 | .8192 |
| $p_{FDR}$ | | | p = .0157 | p = .0044 | - - - |
|  | | * FDR-corrected significant correlation (p<.05)  **fALFF – fractional amplitude of low frequency fluctuations, GMV – gray matter volume** | | | |

| **supplementary file 1e** Fisher’s z transformed Spearman correlations between significant fALFF-neurotransmitter correlations and neuropsychological test data. | | | | | | | | | | | |
| --- | --- | --- | --- | --- | --- | --- | --- | --- | --- | --- | --- |
| **Neurotransmitter** | | **VF** | **BNT** | **MMSE** | **TMTB** | **AES** | **FrSBe Freq** | **FrSBe Dist** | **FrSBe Dist**  **EF** | **CDR-FTLD** | $\boldsymbol{p}_{\boldsymbol{FDR}}$ |
| 5-HT1b | r (Spearman) | .0711 | .0260 | .0633 | -.0638 | .0360 | -.0538 | .1493 | .1168 | -.2186 | --- |
|  | p | .6275 | .8592 | .6625 | .7115 | .8372 | .7623 | .4395 | .5106 | .1491 |  |
| 5-HT2a | r (Spearman) | .0405 | .0540 | .0479 | -.0885 | -.0126 | .0876 | .1787 | .0782 | -.1077 | --- |
|  | p | .7824 | .7127 | .7411 | .6078 | .9426 | .6221 | .3537 | .6601 | .4812 |  |
| GABAa | r (Spearman) | .0369 | -.0341 | .0793 | -.1208 | .0374 | .1562 | .1624 | .0761 | -.1303 | --- |
|  | p | .8012 | .8162 | .5841 | .4828 | .8309 | .3778 | .4000 | .6689 | .3936 |  |
| NET | r (Spearman) | .3719* | .2657 | .3797* | -.1635 | .1437 | .2880 | .3238 | .3521 | -.0838 | p = .0085 |
|  | p | .0085 | .0650 | .0065 | .3407 | .4102 | .0986 | .0866 | .0411 | .5841 |  |
| * FDR-corrected significant correlation (p<.05)  **fALFF – fractional amplitude of low frequency fluctuations, VF – Verbal Fluency, BNT – Boston Naming Test, MMSE – Mini Mental State Exam, TMTB – Trail Making Test B, AES – Apathy Evaluation Scale, FrSBe Freq – Frontal Systems Behavior Scale Frequency Score, FrSBe Dist – Frontal Systems Behavior Scale Distress Score, FrSBe Dist EF – Frontal Systems Behavior Scale Distress Score for Executive Functioning subscale, CDR-FTLD – Clinical Dementia Rating Frontotemporal Lobar Degeneration** | | | | | | | | | | | |

| **supplementary file 1f** Spearman correlations between fALFF Eigenvariates from largest cluster (HC>bvFTD) and neuropsychological test data. | | |
| --- | --- | --- |
| **Eigenvariates correlation with** | **r (Spearman’s rho)** | **p** |
| VF | .2459 | .0885 |
| BNT | .2076 | .1524 |
| MMSE | .2629 | .0651 |
| TMT-B | -.3188 | .0581 |
| AES | .0000 | 1.0000 |
| FrSBe Freq | -.0519 | .7709 |
| FrSBe Dist | .0022 | .9909 |
| FrSBe Dist EF | -.0543 | .7602 |
| CDR-FTLD | -.3385 | .0229 |
| $p_{FDR}$ | --- | |
| **VF – Verbal Fluency, BNT – Boston Naming Test, MMSE – Mini Mental State Exam, TMTB – Trail Making Test B, AES – Apathy Evaluation Scale, FrSBe Freq – Frontal Systems Behavior Scale Frequency Score, FrSBe Dist – Frontal Systems Behavior Scale Distress Score, FrSBe Dist EF – Frontal Systems Behavior Scale Distress Score for Executive Functioning subscale, CDR-FTLD – Clinical Dementia Rating Frontotemporal Lobar Degeneration, FDR – False Discovery Rate** | | |
